# Genetic Structure of Water Chestnut Beetle: Providing Evidence for Origin of Water Chestnut

**DOI:** 10.1371/journal.pone.0159557

**Published:** 2016-07-26

**Authors:** Xiao-Tian Tang, Fu-Shan Zheng, Jing Qin, Ming-Xing Lu, Yu-Zhou Du

**Affiliations:** 1 School of Horticulture and Plant Protection & Institute of Applied Entomology, Yangzhou University, Yangzhou 225009, China; 2 Institute of Shandong River Wetlands, Laiwu, 271100, China; Institute of Vegetables and Flowers, Chinese Academy of Agricultural Sciences, CHINA

## Abstract

Water chestnut beetle (*Galerucella birmanica* Jacoby) is a pest of the water chestnut (*Trapa natans* L.). To analyze the phylogeny and biogeography of the beetle and provide evidence for the origin of *T*. *natans* in China, we conducted this by using three mitochondrial genes (*COI*, *COII* and *Cytb*) and nuclear ITS2 ribosomal DNA of *G*. *birmanica*. As for mtDNA genes, the beetle could be subdivided into three groups: northeastern China (NEC), central-northern-southern China (CC-NC-SC) and southwestern China (SWC) based on SAMOVA, phylogenetic analyses and haplotype networks. But for ITS2, no obvious lineages were obtained but individuals which were from NEC region clustered into one clade, which might be due to sequence conservation of ITS2. Significant genetic variation was observed among the three groups with infrequent gene flow between groups, which may have been restricted due to natural barriers and events in the Late Pleistocene. Based on our analyses of genetic variation in the CC-NC-SC geographical region, the star-like haplotype networks, approximate Bayesian computation, niche modelling and phylogeographic variation of the beetle, we concluded that the beetle population has been lasting in the lower, central reaches of the Yangtze River Basin with its host plant, water chestnut, which is consistent with archaeological records. Moreover, we speculate that the CC-NC-SC population of *G*. *birmanica* may have undergone a period of expansion coincident with domestication of the water chestnut approximately 113,900–126,500 years ago.

## Introduction

Water chestnut beetle, *Galerucella birmanica* Jacoby (Coleoptera: Chrysomelidae), is an important pest of a floating annual aquatic plant, Water chestnut (*Trapa natans* L.), and is relatively host-specific, although it has been known to feed on water shield plants (*Brasenia schreberi* Gmelin) [1, 2]. In China, *G*. *birmanica* is mainly distributed in habitat of *T*. *natans*, such as Yangtze River Basin and Pearl River Basin. In recent decades, many studies have focused on the biology [3–5], ecology [6–8], and control [9]. In America, several studies suggested that *G*. *birmanica* showing promise as potential biological control agents to the invasive plant, *T*. *natans* [10, 11]. However, no further research concentrated on genetic aspects of *G*. *birmanica*, such as genetic diversity, population genetic structure and phylogeography.

The beetle’s host, Water chestnut (*T*. *natans*), was introduced into North America around 1870 and has become invasive in the Northeastern United States and Canada [12]. However, there are distinct and controversial viewpoints about the origin of *T*. *natans*; for example, some scholars consider Africa and Eurasia as the origin of water chestnut, even though it is considered an endangered species in Europe [13–15]. Other researchers suggest Asia as the origin [16–18], particularly parts of China such as the Taihu Basin [10, 19] and lower, central reaches of Yangtze River Basin [20–23]. However, other accounts suggest that *T*. *natans* originated from Europe and Asia [24], and despite this, no genetic evidence give us a clear picture regarding the origin of water chestnut.

The plant–insect co-evolutionary studies have evolved significantly since Ehrlich and Raven (1964) [25] formally introduced the concept of stepwise co-evolution based on butterfly-angiosperm interactions. Interest in co-evolution has encompassed plant-insect, host–parasite, and host–bacteria interactions [26–33]. We hypothesize that there are potential co-evolutionary connections between the water chestnut beetle and water chestnut according to two important points. The first one is the oligophagous character of *G*. *birmanica*. Secondly, the spread of *G*. *birmanica* is directly correlated with distribution of its host plant, *T*. *natans*.

Because mtDNA has some inherent characteristics including haploid uniparental inheritance and the absence of genetic recombination [34]; in addition, ITS regions of rDNA have been developed into a useful tool for many phylogenetic studies [35–37], which could provide additional information to supplement the findings of mtDNA analysis. Therefore, in the present study, on one hand, we integrate phylogenetic and biogeographic methods to reconstruct the population genetic structure, demographic history and molecular phylogeny of water chestnut beetle using three mtDNA genes (*COI*, *COII* and *Cytb*) and nuclear ribosomal internal transcribed spacer 2 (ITS2) region. On the other hand, our study may provide indirect evidence for the geographical origin of water chestnut, which has long been debated, based on genetic structure of the water chestnut beetle.

## Materials and Methods

### Sample collection and DNA extraction

**Fig 1:**
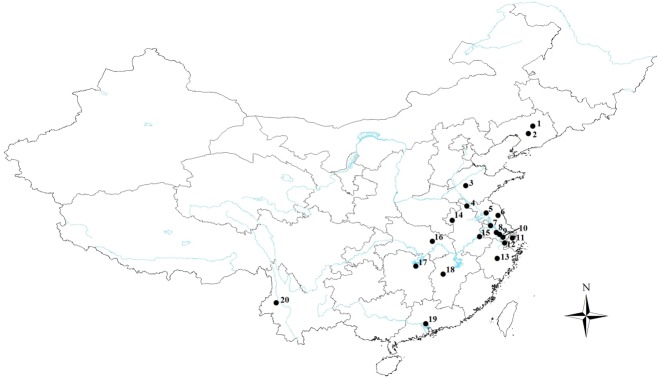
Sampling sites of *G*. *birmanica* in China. Maps were created using Esri’s ArcGIS platform (http://www.esri.com/software/arcgis). Numbers represent the following geographical locations (city, province): 1, Shenyang, Liaoning; 2, Anshan, Liaoning; 3, Taian, Shandong; 4, Xuzhou, Jiangsu; 5, Huaian, Jiangsu; 6, Yancheng, Jiangsu; 7, Yangzhou, Jiangsu; 8, Changzhou, Jiangsu; 9, Wuxi, Jiangsu; 10, Suzhou, Jiangsu; 11, Shanghai; 12, Jiaxing, Zhejiang; 13, Yiwu, Zhejiang; 14, Fuyang, Anhui; 15, Wuhu, Anhui; 16, Xiaogan, Hubei; 17, Yiyang, Hunan; 18, Xinyu, Jiangxi; 19, Guangzhou, Guangdong; and 20, Baoshan, Yunnan.

In this study, we collected specimens of *G*. *birmanica* and *G*. *grisescens* (outgroup) on *T*. *natans* from 20 populations in ten provinces and one city. This included most of the areas where water chestnut occurs in China including Liaoning, Shandong, Jiangsu, Zhejiang, Anhui, Hubei, Hunan, Jiangxi, Guangdong, Yunnan and Shanghai (Fig 1; S1 Table). We declare that no specific permissions were required for these activities and that the field studies did not involve endangered or protected species. It is noteworthy that the sampling areas covered five zoogeographical regions, including northeastern China (NEC), northern China (NC), southwestern China (SWC), central China (CC) and southern China (SC). Specimens were preserved in 100% ethanol at -20°C until processing. Genomic DNA was then extracted from the thorax of individual beetles using a cetyltrimethyl ammonium bromide (CTAB) extraction protocol.

### PCR amplification and sequencing

Three fragments of the mitochondrial genome (837, 681, and 462 bp of *COI*, *COII* and *Cytb*, respectively) were amplified from individual beetles using primer pairs C1-J-2183 (5’-CAACATTTATTTTGATTTTTTGG) and TL2-N-3014 (5’-TCCAATGCACTAATCTGCCATATT) [38], COII-F (5’-TAATATGGCAGATTAGTGCATTGGA) and COII-R (5’-GAGACCATTACTTGCTTTCAGTCATCT)[39], and Cytb-F (5’-TATGTACTACCATGAGGACAAATATC) and Cytb-R (5’-ATTACACCTCCTAATTTATTAGGAAT) [38], respectively. PCR reactions were carried out in 50 μL aliquots containing 100 ng extracted DNA, 5 μL 10× Taq buffer, 4 μL 25 mmol/L MgCl_2_, 4 μL 25 mmol/L dNTPs, 1 μL 20 μmol/L primers, 0.4 μL rTaq DNA polymerase (5U/μL), and ultra-pure water. PCR was conducted using the following conditions: 94°C for 4 min; 35 cycles of 94°C for 50 s, Tm for 50 s, 72°C for 1 min, and a final extension step of 72°C for 10 min. About 680 bp fragment of 5.8S-ITS2-28S region was amplified with primers: ITS2-F (5’-GCATCGATGAAGAACGCAGC) and ITS2-R (5’-TCCTCCGCTTATTGATATGC) [40]. All amplification products above were purified using the AxyPrep™ DNA Gel Extraction Kit (Axygen, Union City, CA, USA), cloned into pGEM-T Easy Vector (Promega), and then transformed into *Escherichia coli* DH5α cells. Finally, a total of 156 positive clones (three repeats) were picked and sequenced by IGE Biotechnology Co., Ltd (Guangzhou, China) and Sangon Biotechnology Co., Ltd (Shanghai, China).

### Statistical analysis

The three mtDNA gene sequences were initially aligned using CLUSTAL X v. 1.83 [41] and then manually aligned. Haplotype diversity (H), nucleotide diversity (π) and the average number of nucleotide differences (K) were calculated using DnaSP v. 5.0 [42].

Spatial analysis of molecular variance was performed using SAMOVA v. 1.0 [43] to identify population groups. One hundred simulated annealing processes were used for each value of K (number of groups). The most supported number of groups (K) was determined by repeating the analysis with K ranging from 2 to 10 and selecting the subdivision scheme associated with the highest F_CT_. Analyses of molecular variance (AMOVA) were performed using Arlequin v. 3.5 [44] based on the groups inferred by SAMOVA analysis. F-statistic (F_ST_) values were also calculated. Moreover, using the formula F_ST_ *=* 1/ (1 + 2N_m_), which is specific for organelle genetic data [45], we derived the value for gene flow (N_m_). Phylogenetic trees were constructed using MrBayes v. 3.2.1 [46] and a PHYML online web server [47–48]; *G*. *grisescens* served as the outgroup. The best fit model for nucleotide alignments was determined by Modeltest 3.7 [49]. According to the Akaike information criterion, the GTR + I + G paradigm was the best model for analysis using nucleotide alignments. For analysis by Bayesian inference (BI), nucleotide alignments were constructed using the MrBayes program with 1,000,000 generations and with the first 25% discarded as burn-in. Maximum likelihood (ML) analysis was performed using the following conditions: the proportion of invariable sites was “estimated,” the number of substitution rate categories was four; the gamma distribution parameter was “estimated,” and the starting tree was a BIONJ distance-based tree. Tree data were visualized and edited using FigTree v. 1.3.1 [50]. Median-joining networks of haplotypes of the three genes were constructed using NETWORK v. 4.6 [51] and used to infer evolutionary relationships among haplotypes.

**Fig 2:**
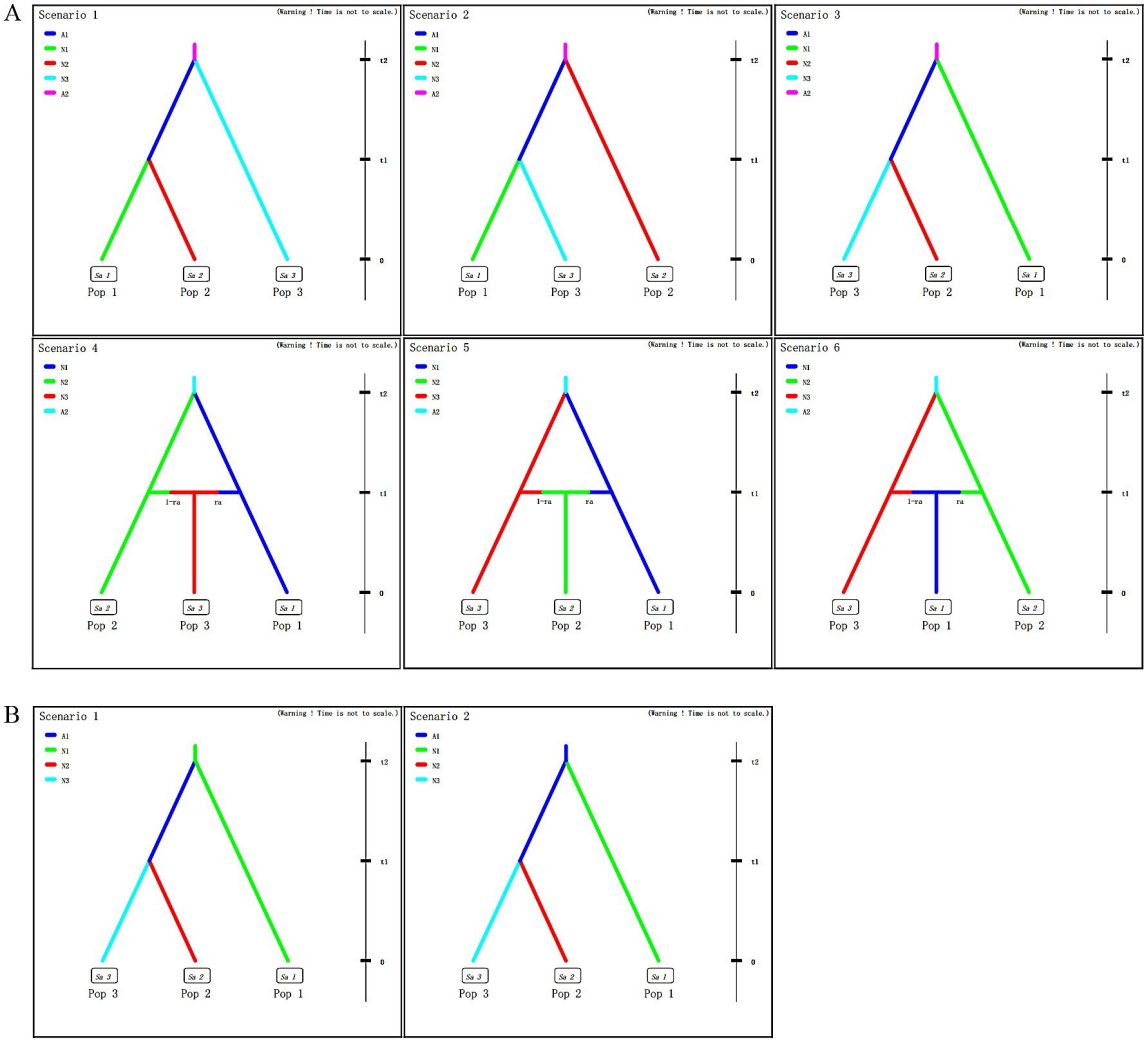
Scenarios for the DIY ABC analyses, which was designed to infer the origin of *G*. *birmanica*. A, Six scenarios showing relationships of three groups regarding the variation in population size and the split and admixture events. B, Two scenarios showing the origin of *G*. *birmanica*.

The software DIYABC version 2.0 [52] was used to compare different competing scenarios regarding the ancestral populations of *G*. *birmanica*. Step 1, a total of six scenarios were considered regarding the variation in population size and the split and admixture events (Fig 2A); Step 2, two competing scenarios based on the scenario that northeastern China (NEC) and southwestern China (SWC) split from central-northern-southern China (CC-NC-SC) were analyzed, regarding the source of the entire China populations (Fig 2B).

We used ecological niche modelling to predict the geographic distribution of climatically suitable habitats for *G*. *birmanica* within our study area and analyze whether climatic stability and current and past climate conditions are responsible for observed patterns of genetic diversity and structure using MaxEnt [53]. We obtained bioclimatic data layers for current (1950–2000), last glacial maximum (LGM) and last interglacial (LIG) conditions from the WorldClim database (http://worldclim.org/current.htm) [54]. The random test percentage was set to 25%, and the Jackknife procedure was used to estimate the contribution of each variable based on performance of the model. The area under the curve (AUC) value was calculated for model validation; AUC reflects the model’s ability to distinguish between present records and random background points. AUC values ranged from 0.5 (not different from a randomly-selected predictive distribution) to 1 (with perfect predictive ability). Models having AUC values >0.9 were considered to have very good, >0.8 good, and >0.7 useful discrimination abilities [55]. The final map was visualized and processed using the ArcGIS platform (http://www.esri.com/software/arcgis).

The demographic history of all populations (pooled) and individual population groups identified by SAMOVA analysis was studied using mismatch distributions in ARLEQUIN. Tajima’s *D* and Fu’s *F*_*S*_ tests were used to test for neutrality. Population expansion time (τ) and the sum of squared deviation (SSD) between observed and expected mismatch distributions were similarly calculated. All parameters were evaluated based on 1,000 bootstrap replicates.

## Results

### Genetic diversity

The length of aligned sequences of *COI*, *COII* and *Cytb* genes was 837, 681 and 462 bp, respectively. Among individuals from 20 populations, 33 *COI*, 23 *COII* and 20 *Cytb* haplotypes were identified; these were submitted to GenBank as accession numbers EF512802-EF512833, EF512778-EF512800 and EF512757-EF512776, respectively. The average pairwise sequence divergence among haplotypes of *COI*, *COII* and *Cytb* was 0.965, 0.883 and 0.947, respectively (Table 1). Nucleotide diversity (π) over all populations was 0.01111, 0.01147 and 0.01344, respectively. Interestingly, there was a larger genetic variation in the CC-NC-SC geographic region as compared to other areas of China (see bold font, Table 1).

**Table 1:** Parameters of genetic diversity and demographic analysis.

Gene	Group	H	π	τ	*D*	*Fs*	SSD
COI	All	0.965	0.01111	1.764	-0.2265	-12.5864**	0.0145
	NEC	0.000	0.00000	0.000	0.0000	0.0000	-
	SWC	0.867	0.00175	1.688	0.6003	-1.0719	0.0155
	CC-NC-SC	**0.969**	**0.00498**	4.387	-1.2738	-23.1881**	0.0019
COII	All	0.883	0.01147	0.000	-0.2006	-4.1877	0.8454**
	NEC	0.476	0.00070	0.717	0.5590	0.5887	0.0172
	SWC	0.733	0.00137	1.186	0.3106	-0.3041	0.0310
	CC-NC-SC	**0.805**	**0.00320**	0.902	-1.9292**	-13.1469**	0.0013
Cytb	All	0.947	0.01344	10.814	-0.2584	-5.4544*	0.0096
	NEC	0.600	0.00188	1.320	-0.0500	-0.4268	0.0068
	SWC	0.400	0.00087	0.562	-0.8165	0.0902	0.0072
	CC-NC-SC	**0.929**	**0.00611**	2.689	-0.9796	-8.2840**	0.0032
ITS2	All	0.660	0.00234	2.020	-2.6656**	-27.2321**	0.0008
	NEC	0.644	0.00209	1.764	-0.58152	-1.16409	0.0668*
	SWC	0.400	0.00082	0.562	-0.81650	0.09021	0.0072
	CC-NC-SC	0.635	0.00222	1.480	-2.6822**	-27.1542**	0.0017

H: Haplotype diversity; π: nucleotide diversity; τ: population expansion time; *D*: Tajima’s *D*; *Fs*: Fu’s *Fs*; SSD: sum of squared deviations between observed and expected mismatch distribution under a sudden expansion model; bold indicates there is a larger genetic variation in CC-NC-SC region than in other regions

*P<0.05

** *P*<0.01.

Moreover, after trimming of the sequences, 488–492 bp fragments were obtained for ITS2 region (with four nucleotides INDEL block). 34 unique sequences generated during this research were submitted to GenBank (accession numbers: KR401224-KR401257). However, ITS2 haplotype and nucleotide diversity were relatively low (H = 0.660 and π = 0.00234).

We also calculated the number of unique haplotypes (n), haplotype diversity (H), nucleotide diversity (π) and the average number of nucleotide differences (K) for each population (S2 Table). Overall, our statistics suggest that entire populations of *G*. *birmanica* retain high levels of genetic diversity.

### Population genetic structure and phylogenetic analysis

**Fig 3:**
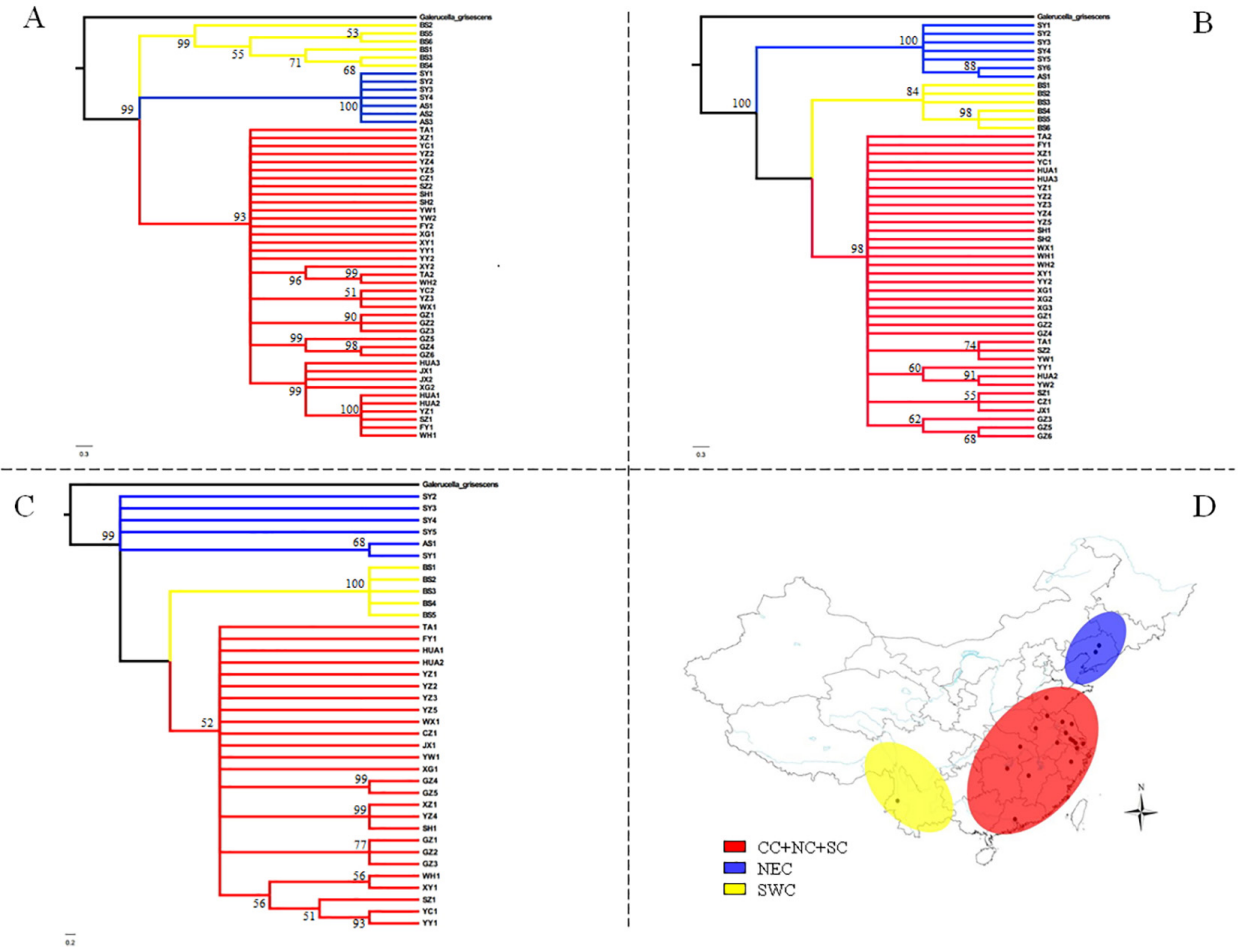
Bayesian phylograms based on sequences obtained from *G*. *birmanica* samples. Sequence data are shown for *COI* (A), *COII* (B); and (C) *Cytb*. (D) Geographical distribution of the three groups. Abbreviations: CC-NC-SC (central-northern-southern China); NEC, northeastern China (NEC), and SWC, southwestern China.

**Fig 4:**
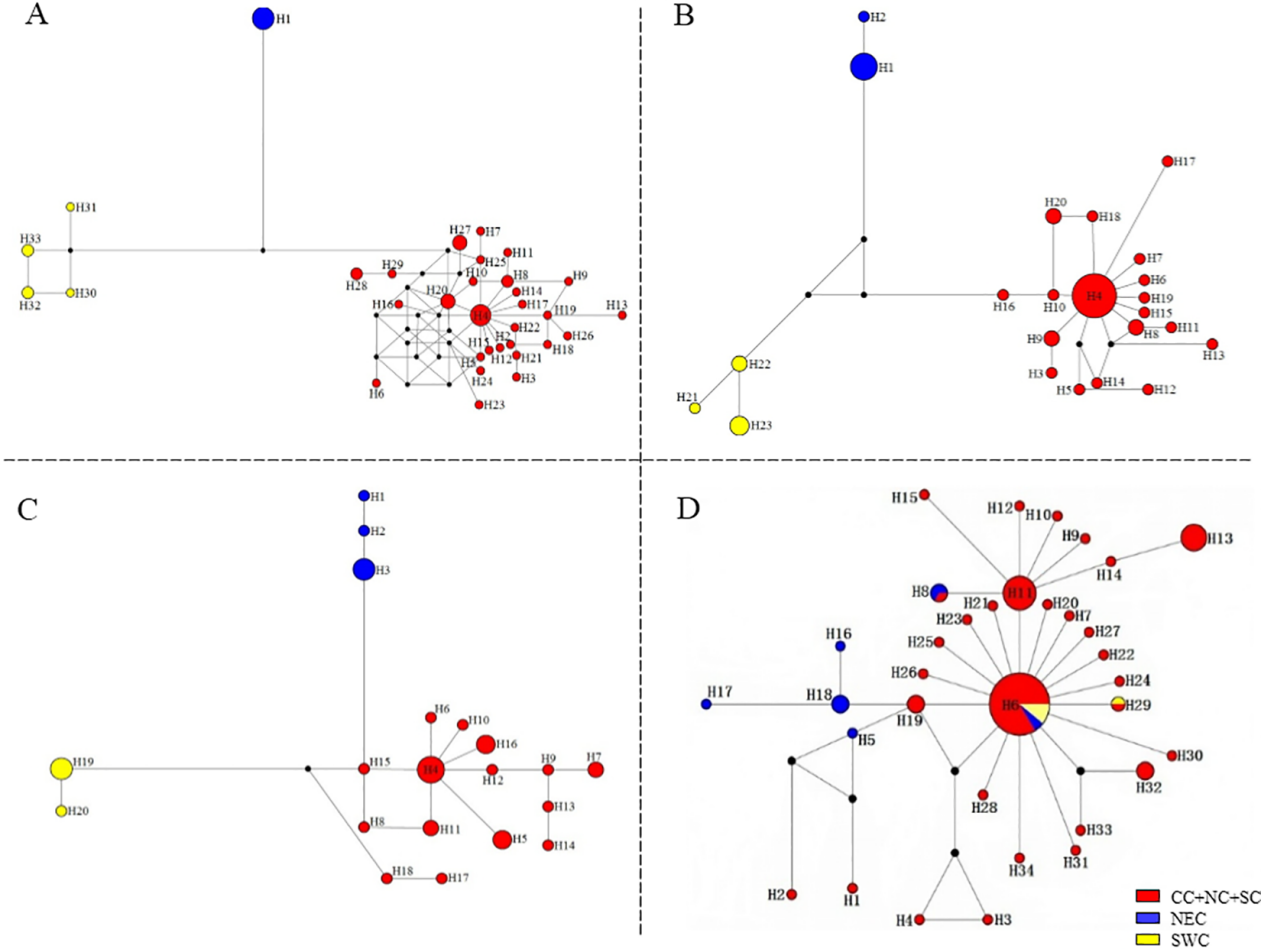
Median-joining (MJ) networks of *G*. *birmanica* haplotypes based on analysis of the following genes (A) *COI*; (B) *COII*; (C) *Cytb* and (D) ITS2 region. The sizes of circles are proportional to the number of individuals in the data point. Abbreviations: CC-NC-SC (central-northern-southern China); NEC, northeastern China (NEC), and SWC, southwestern China.

**Fig 5:**
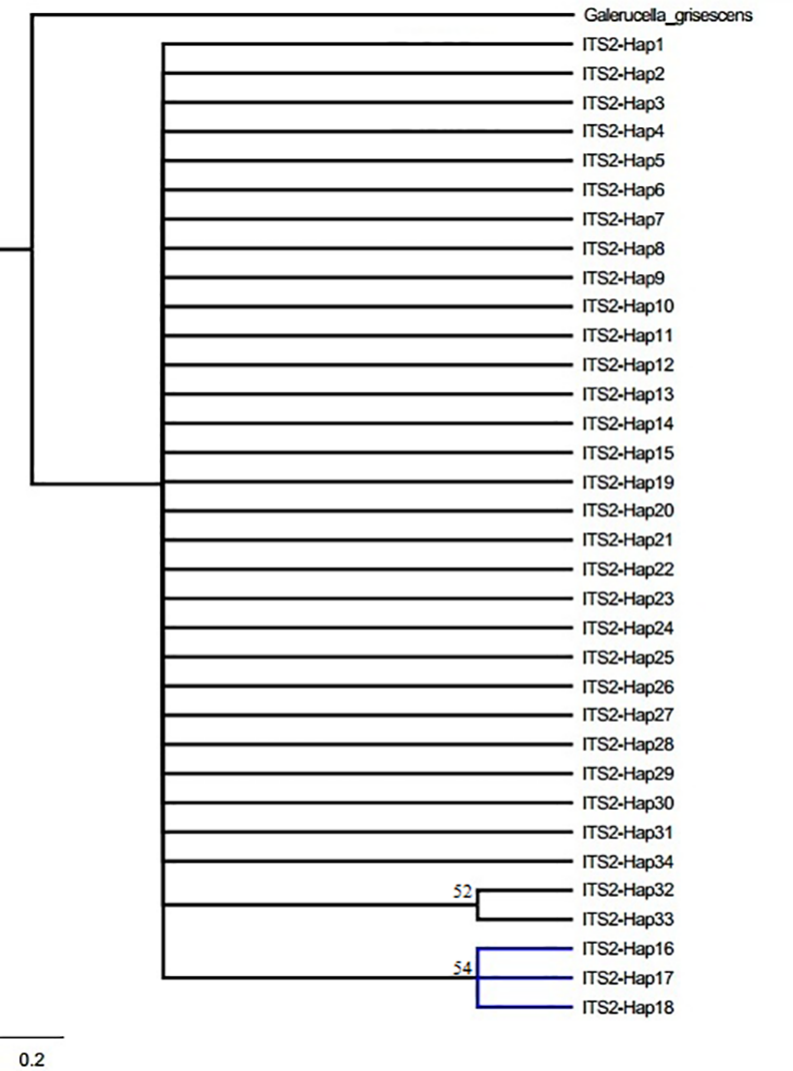
Bayesian phylograms based on ITS2 region sequences obtained from *G*. *birmanica* samples. Blue indicates haplotypes from northeastern China (NEC) region.

When F_CT_ values were analyzed by SAMOVA, three population groups were suggested as the optimal number for *COII* (F_CT_ = 0.88007), *Cytb* (F_CT_ = 0.76822) and ITS2 (F_CT_ = 0.31787); however, five groups were suggested when *COI* was considered (F_CT_ = 0.78653) (Table 2). Populations from the 20 geographical locations formed three clusters as follows: SY and AS; TA, XZ, HUA, YC, YZ, CZ, WX, SZ, SH, JX, YW, FY, WH, XG, YY and XY; and BS (codes represent geographical locations; see S1 Table). The three clusters correspond to three geographically distinct regions that we refer to as northeastern China (NEC), central-northern-southern China (CC-NC-SC), and southwestern China (SWC), which agreed with the phylogenetic trees constructed with Bayesian and ML methods and the three mtDNA genes. The phylogenetic trees had similar topology using the two methods; consequently, only phylograms derived using Bayesian analysis are shown (Fig 3). Interestingly, networks constructed using median-joining corroborated the assignment of haplotypes into one of the three clades (NEC, CC-NC-SC and SWC) (Fig 4). However, as for phylogenetic tree based on ITS2 ribosomal DNA, no obvious lineages were obtained but with haplotypes which were from NEC region clustered into one clade (Fig 5).

**Table 2:** AMOVA results comparing genetic variation in *Galerucella birmanica* collected from 20 localities among the three geographical regions.

Gene analyzed	Source of variation	d.f.	Sum of squares	Variance components	Percentage of variation	Fixation indices	*P*-value
COI	Among groups	4	172.577	5.43111 Va	78.65	F_CT_ = 0.78653	0.00000±0.00000
	Among populations within groups	15	28.200	0.33966 Vb	4.92	F_SC_ = 0.23043	0.00000±0.00000
	Within populations	32	36.300	1.13437 Vc	16.43	F_ST_ = 0.83572	0.00000±0.00000
COII	Among groups	2	145.487	6.85924 Va	88.01	F_CT_ = 0.88007	0.00000±0.00000
	Among populations within groups	17	17.601	0.09683 Vb	1.24	F_SC_ = 0.10359	0.00000±0.00000
	Within populations	29	24.300	0.83793 Vc	10.75	F_ST_ = 0.89249	0.00000±0.00000
Cytb	Among groups	2	74.014	4.00512 Va	76.82	F_CT_ = 0.76822	0.00000±0.00000
	Among populations within groups	17	24.351	0.47308 Vb	9.07	F_SC_ = 0.39150	0.00000±0.00000
	Within populations	17	12.500	0.73529 Vc	14.10	F_ST_ = 0.85896	0.00000±0.00000
ITS2	Among groups	2	6.432	0.09878 Va	8.64	F_CT_ = 0.31787	0.18573±0.01021
	Among populations within groups	16	30.663	0.21802 Vb	19.07	F_SC_ = 0.20876	0.00000±0.00000
	Within populations	76	62.800	0.82632 Vc	72.29	F_ST_ = 0.37713	0.00000±0.00000

Analyses of molecular variance (AMOVA) revealed that most (*COI*, 78.65%; *COII*, 88.01%; *Cytb*, 76.82%) of the variation was distributed between groups (Table 2). Genetic differentiation was limited among populations within groups (*COI*, 4.92%; *COII*, 1.24%; *Cytb*, 9.07%) and within individual populations (*COI*, 16.43%; *COII*, 10.75%; *Cytb*, 14.10%) (Table 2). Fixation indices that indicate genetic differentiation, including F_CT_ (between groups), F_SC_ (among populations within groups) and F_ST_ (within populations) were all highly significant (*P*<0.01) for the three mtDNA genes (Table 2). The hierarchical AMOVA was also conducted for ITS2 region. Unlike mtDNA genes, the main genetic variation attributed to within populations (72.29%, Table 2).

Median-joining networks had star-like conformations with limited substructures (Fig 4). For three mtDNA genes, members of haplotype 4, which derived from beetle populations inhabiting the lower, central Yangtze River Basin in the CC-NC-SC region (e.g. YZ, SZ, XZ, HUA, YC, SH, WH and FY), were considered to be ancestral since they had a central position in the networks (Fig 4A, 4B and 4C). The remaining haplotypes formed star-like topologies that were consistent with recent population expansion [56, 57]; furthermore, three clades (marked in red, yellow and blue, see Fig 4) were generally present in each network. The three clades represent three geographically-distinct regions with no shared haplotypes, suggesting that the populations differentiated within the three different regions. However, no obvious lineages were obtained in the median joining networking of the ITS2 ribotypes (Fig 4D). We must note that the network shows few mutational steps and also displays a star like pattern, where the most common ribotype (Hap 6) lied at the star’s center and derivatives were connected to it by short branches (Fig 4D). Most of haplotype 6s also derived from the lower, central Yangtze River Basin in the CC-NC-SC region but with few from NEC and SWC regions. Haplotype 11 as the secondary common haplotype were all from Taihu Basin in lower Yangtze River Basin including SZ and CZ populations.

F_ST_ values for the three regions were 0.732–0.963 for *COI*, 0.826–0.957 for *COII*, 0.728–0.952 for *Cytb*, but -0.025–0.200 for ITS2 (Table 3). However, the N_m_ values representing gene flow were low: 0.019–0.183 for *COI*; 0.022–0.105 for *COII*; 0.025–0.187 for *CytB*; but more than 2.000 for ITS2 (Table 3). We also provide F_ST_ and N_m_ values for 20 populations in S3–S5 Tables. These data indicate a very low level of gene flow between populations from different regions, whereas a high level of gene flow was observed between population clusters residing in the CC-NC-SC region.

**Table 3:** Estimates of F_ST_ and gene flow (N_m_) for pairs of the three regions.

	NEC	CC-NC-SC	SWC
NEC		0.105, 0.056, 0.187, 2.038	0.019, 0.022, 0.025, 2.000
CC-NC-SC	0.826, 0.899, 0.728, 0.197		0.183, 0.105, 0.134, Inf
SWC	0.963, 0.957, 0.952, 0.200	0.732, 0.826, 0.788, -0.025	

The data above the diagonal are N_m_; the data below the diagonal are F_ST_; Data from left to right represent COI, COII, Cytb genes and ITS2 region, respectively.

### Scenario testing

In the scenario testing analyses of step 1, the scenario with the highest likelihood was scenario 3 (logistic regression of COI, COII and Cytb is 0.7377 [95% CI: 0.4770–0.9983], 0.5784 [95% CI: 0.3088–0.8479], 0.3613 [95% CI: 0.2848, 0.4378], respectively) (Fig 2A; Table 4), indicating a split of NEC and SWC from CC-NC-SC. In step 2, the scenario testing revealed that samples from NEC and SWC region most likely originated from CC-NC-SC region (Fig 2B; Table 4).

**Table 4:** Description of the scenarios used in the approximate Bayesian.

	COI			COII			Cytb		
Steps	Scenario	Posterior probability	95% CI (lower-upper)	Scenario	Posterior probability	95% CI (lower-upper)	Scenario	Posterior probability	95% CI (lower-upper)
Step1	1	0.0004	(0.0000, 0.8308)	1	0.0555	(0.0000, 0.4407)	1	0.1456	(0.0873, 0.2039)
	2	0.0057	(0.0000, 0.8326)	2	0.0199	(0.0000, 0.4211)	2	0.0498	(0.0000, 0.1109)
	3	0.7377	(0.4770, 0.9983)	3	0.5784	(0.3088, 0.8479)	3	0.3613	(0.2848, 0.4378)
	4	0.1872	(0.0000, 0.9068)	4	0.1436	(0.0000, 0.4952)	4	0.1170	(0.0611, 0.1729)
	5	0.0652	(0.0000, 0.8509)	5	0.0908	(0.0000, 0.4590)	5	0.2045	(0.1441, 0.2649)
	6	0.0038	(0.0000, 0.9628)	6	0.1118	(0.0000, 0.6211)	6	0.1218	(0.0241, 0.2195)
Step2	1	0.6342	(0.4240, 0.8444)	1	0.5630	(0.3992, 0.7269)	1	0.5965	(0.4562, 0.7367)
	2	0.3658	(0.1556, 0.5760)	2	0.4370	(0.2731, 0.6008)	2	0.4035	(0.2633, 0.5438)

Computation analysis in DIYABC to test the source and the differentiation time among species. The relative posterior probabilities and 95% confidence intervals for each scenario were computed via the logistic regression on 1% of the closest data sets to the observed data.

### Niche modelling

**Fig 6:**
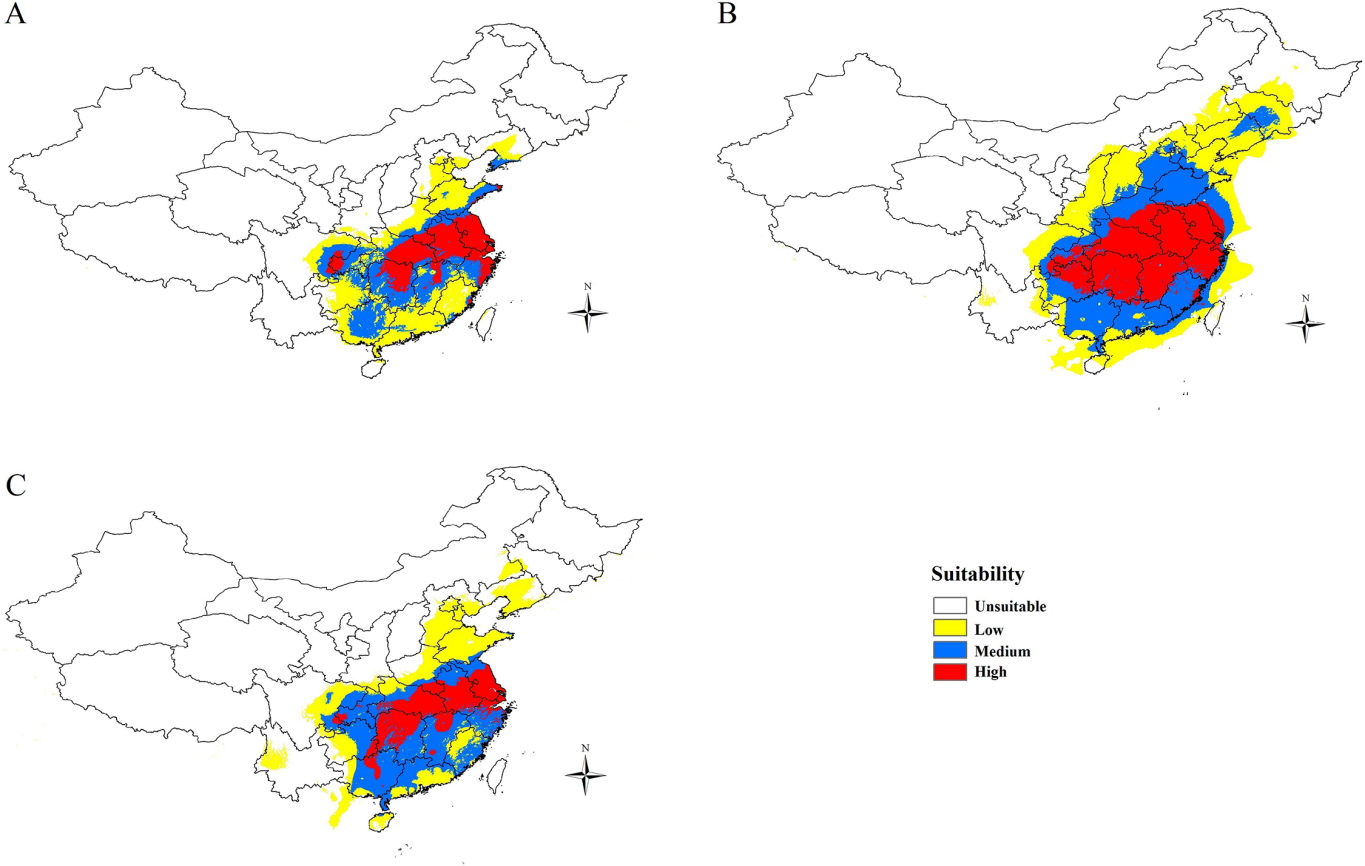
Potential distribution of *G*. *birmanica* under (A) current, (B) last glacial maximum (LGM) and (C) last interglacial (LIG) climate conditions. Maps were created using Esri’s ArcGIS platform (http://www.esri.com/software/arcgis).

The area under the curve (AUC) for the test data was 0.991–0.994, indicating a high fit of the modelled and observed distribution [53, 58]. The MaxEnt distribution models indicates that *G*. *birmanica* populations had a larger distribution during the LGM with a range contraction between the LGM and its current distribution (Fig 6A and 6B). What’s more, the LIG distribution mirrored current distribution (Fig 6C). As a whole, the region of the lower, central reaches of the Yangtze River Basin was predicted to be highly suitable for *G*. *birmanica*, which may potentially suggest that this region acted as refugia during glacial period (Fig 6). In addition, the Jackknife evaluation indicated that precipitation during the warmest quarter (Bioclim 18) was the main factor influencing the distribution of *G*. *birmanica*.

### Demographic history

For mtDNA genes, neutrality tests using Tajima’s *D* statistical test generally resulted in negative values with no significance; an exception was *COII* in the CC-NC-SC region (-1.9292, *P* < 0.01, Table 1). When Fu’s *F*_*S*_ test was utilized, samples from the CC-NC-SC region deviated significantly from neutrality, suggesting that the CC-NC-SC region is responsible for population disequilibrium (Table 1). As for ITS2 region, significantly negative values of Tajima’s *D* (-2.6656, *P* < 0.01) and Fu’s *F*_*S*_ (-27.2321, *P* < 0.01) indicated that the whole set of *G*. *birmanica* samples studied here did not fit a simple model of neutral evolution.

**Fig 7:**
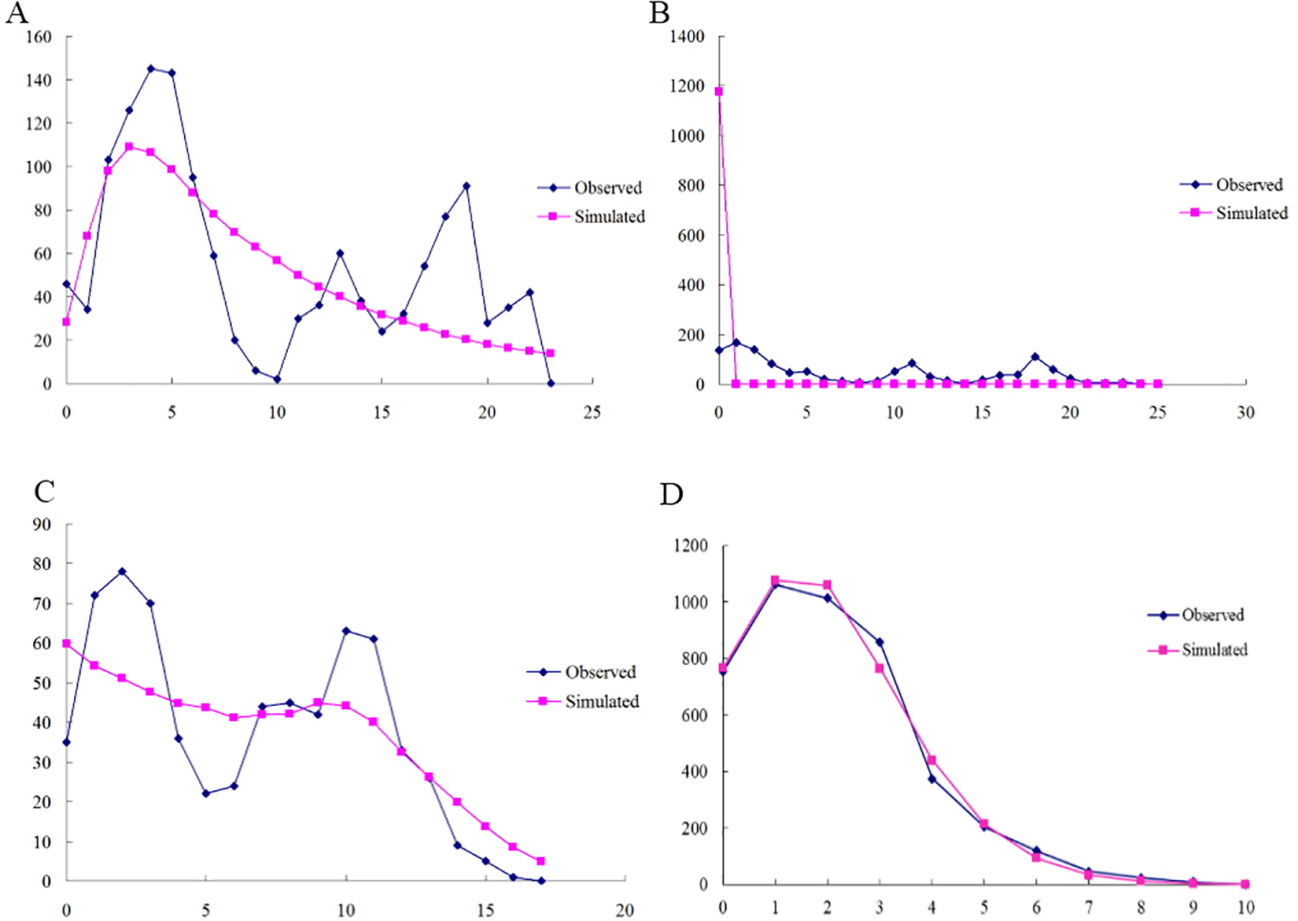
Observed and simulated mismatch distributions of entire samples. Sequence data are shown for (A) *COI*; (B) *COII*; (C) *Cytb* and (D) ITS2 region. The horizontal axis represents the number of pairwise differences, and the vertical axis shows relative frequency.

**Fig 8:**
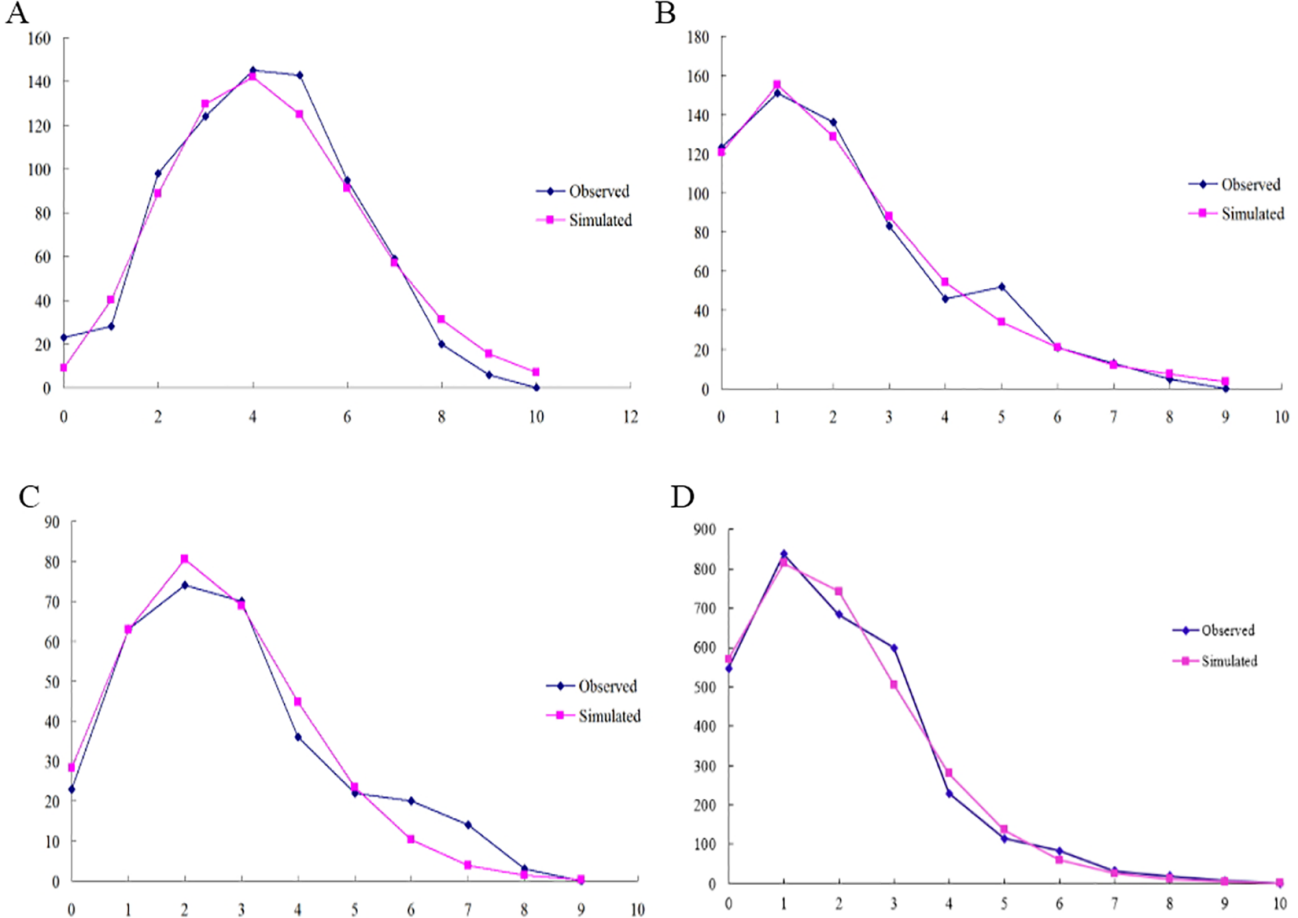
Observed and simulated mismatch distributions of the CC-NC-SC group. Sequence data are shown for (A) *COI*; (B) *COII*; (C) *Cytb* and (D) ITS2 region. The horizontal axis represents the number of pairwise differences, and the vertical axis represents the relative frequency.

Since most of the populations belonged to the CC-NC-SC geographical region, we constructed mismatch distributions for all regions combined and the CC-NC-SC region separately based on mtDNA gene and ITS2 region. Surprisingly, we found that the mismatch distribution of all 20 populations pooled together as well as of the CC-NC-SC region only was distinctly diverse based on mtDNA gene. When the *F*_*S*_ test was utilized, significant, negative values were obtained for *COI (*-12.5864, *P* < 0.01) and *Cytb* (-5.4544, *P* < 0.05) as well as multimodal curves were obtained for all groups (Fig 7), indicating no recent population expansion events for all populations. Analysis of the CC-NC-SC region separately using the *F*_*S*_ statistic showed significant negative values of neutrality for all three genes (*COI*, -23.1881, *P* < 0.01; *COII*, -13.1469, *P* < 0.01; and *Cytb*, -8.2840, *P* < 0.01) and a unimodal curve, which demonstrates population expansion within the CC-NC-SC region (Fig 8). However, The mismatch distribution of all *G*. *birmanica* populations pooled together as well as of the CC-NC-SC region only were distinctly unimodal based on ITS2 region (Figs 7 and 8), coupled with significantly negative values of Tajima’s *D* and Fu’s *F*_*S*_, suggesting recent population expansion events.

Besides, positive *D* and *F*_*S*_ values of mtDNA genes as well as no significant value of *D* and *F*_*S*_ in the NEC and SWC regions, such as *D* = 0.6003 in SWC (*COI*) and *F*_*S*_ = 0.5887 in NEC (*COII*) may indicate recent population bottlenecks in the two regions (Table 1). The sum of squared deviation (SSD) did not exclude the sudden expansion model due to a no significant difference from the expected distribution (*P* > 0.05).

The τ value (mean of population expansion time) of CC-NC-SC region that was estimated for the sudden expansion model was approximately 4.387, 2.689 and 1.480 mutation units for *COI*, *Cytb* and ITS2 ribosomal DNA, respectively, but only 0.902 for *COII* (Table 1). The τ parameter for *Cytb* (all samples) was 10.814, whereas τ for *COII* was 0.000.

## Discussion

As mentioned above, greater genetic differentiation was observed between the three groups versus between populations within groups and within populations based on mtDNA genes but relatively lower revealed by ITS2 ribosomal DNA (Table 2). The genetic differentiation may be attributed to geographical isolation and the nonmigratory behavior of the water chestnut beetle. With respect to geographical isolation, the genetic differentiation of the NEC and CC-NC-SC regions may be related to the geography of the Yellow River and Yanshan Mountain; whereas the CC-NC-SC and SWC regions are demarcated by the Wu Mountain and Xuefeng Mountain, respectively. Concerning its nonmigratory character, *G*. *birmanica* has limited dispersal ability; e.g., normally 15–20 m under favorable conditions [4]. It is also important to consider that a greater degree of genetic diversity may be related to a longer evolutionary history. High mitochondrial haplotypic diversity contrasting with lower of the ITS2 was also reported in many insects, such as *Anopheles funestus* [59] and mite species [60], which might due to low selective pressure or concerted evolution on ITS region [61, 62].

Based on analyses of three mtDNA sequences using AMOVA, phylogenetic tests, and haplotyping, we conclude that *G*. *birmanica* has three genetically-diverse, geographically-localized clades in China; these are the NEC, CC-NC-SC and SWC clades. According to AMOVA, more than 75% of the variability was observed between these three regions with some differences arising between populations based on the three mtDNA genes; this indicates that the three groups have infrequent gene flow and are distinct entities. Thus it is tempting to hypothesize that the natural barriers present in the study area (Yellow River and the Yanshan Mountain, Wu Mountain, and Xuefeng Mountain) may deter or limit gene flow; whereas gene flow in the CC-NC-SC region was frequent. Similar results have been obtained for the *Chilo suppressalis* [63], where rivers and mountains function as effective barriers to gene flow. More importantly, this pattern may be related to events that occurred in the Late Pleistocene (75,000–130,000 years ago), when geographic events occurred that caused separate populations to arise within species [64]. However, no obvious lineages obtained and most of the variability was observed were within populations based on ITS2 ribosomal DNA, which may be due to its conserved character.

Based on a proposed standard rate of 2.3% divergence per million years (My) for the insect mitochondrial genome [65] (But rate of divergence for ITS region was unknown), the coalescence time (equated with the onset of demographic or range expansion) for *COI*, *COII*, and *Cytb* in the CC-NC-SC region is about 113,900 (τ = 4.387 mutation units), 28,800 (τ = 0.902 mutation units) and 126,500 years (τ = 2.689 mutation units), respectively. Due to the lower number of sample sites in the NEC and SWC regions and reduced haplotype diversity, divergence times in these two regions could not be accurately predicted.

Analyses of the *F*_*S*_ statistics in the three groups and mismatch distribution of the CC-NC-SC region suggested that the CC-NC-SC group may have undergone population expansion, whereas the NEC and SWC regions may have experienced recent bottlenecks. This may have occurred because the *G*. *birmanica* population did not adapt to the local habitat and climate changes, especially in the colder NEC region. The contention that the beetle species underwent population expansion is consistent with the observation that median-joining networks for the three mtDNA genes and ITS2 region have a distinct star-like structure, which is typical of demographic expansion. This structure is particularly obvious in the CC-NC-SC group, and haplotypes from the NEC and SWC regions are derived from a subset of the CC-NC-SC group that is the ancient mtDNA haplotype4s (H4s) and ITS2 haplotype 6 and 11 are derived from this group. In addition, the scenario tests based on ABC method revealed that the CC-NC-SC area is the most likely origin of the beetle in China. Coupled with this area acted as refugia during glacial period, therefore, we speculate that the water chestnut beetle originated in the lower, central reaches of the Yangtze River Basin. What’s more, populations at the center of origin generally exhibit the greatest variation, which is consistent with the perspective that ancestral populations possess higher genetic diversity than derived populations [66]. The larger genetic variation in the CC-NC-SC geographical region also supported our speculation.

**Fig 9:**
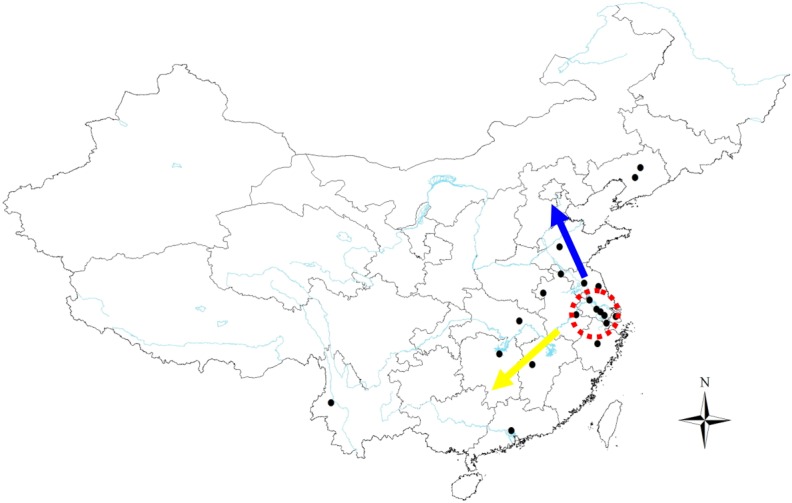
Inferred dispersal route of the water chestnut beetle, *G*. *birmanica* (arrow shows the direction of dispersal from the proposed origin). Maps were created using Esri’s ArcGIS platform (http://www.esri.com/software/arcgis).

Water chestnut beetles are likely to have co-evolved with the host plant, water chestnut; thus, it has been suggested that the insect and pest share a common origin, which we speculate is located in the lower, central region of the Yangtze River Basin. Recent studies suggest that the Yangtze Valley is a potential center of origin for domesticated, aquatic vegetables [22, 23, 67], rice [68, 69] and dog [70, 71]. As pointed out in the introduction, the origin of water chestnut is unclear and both Asia and Europe are potential geographical origins. There are numerous species and variants of *T*. *natans* in Asia, including wild *T*. *natans*, *T*. *japonicum*, domesticated *T*. *bicornis* and *T*. *bispinosa*. In China, 15 species and variants of water chestnut have been identified, whereas only wild *T*. *natans* occurs in Europe and North America. Furthermore, water chestnut is now considered an endangered species in Europe and Russia [14]. These differences in biogeography compel us to speculate that the introduction or occurrence of water chestnut is more recent in Europe. More importantly, analysis of Neolithic (3,300 to 2,800 BC) sediment collected from archeological sites in the village of Pannala, southern Finland, revealed that the dominant aquatic plant in lake basins was water chestnut, along with species of *Potamogeton*, *Nymphaea*, and *Nuphar*, which also have a floating leaf habit [72]. However, archaeological evidence for water chestnut in Zhejiang, China, was documented 20,000–30,000 years ago based on geological exploration 78 meters underground [22]. It is also important to mention the discovery of charred water chestnuts at the Hemudu archaeological site in the 1970s as these were estimated to be over 7,000 years old [21]. A similar finding was made at the Majiabang site where carbonized water chestnuts approximately 6,000 years old were discovered [20]. Furthermore, the Taihu Basin in the lower, central reaches of the Yangtze River is regarded by some researchers as a center of origin for water chestnut [10, 19]. In Chinese history, reference to water chestnuts occurs many times in ancient literature; e.g. “Zhou Li” (300 BC) and “Guo Yu” (400 BC). Poems about water chestnuts were also popular in ancient China, including the Tang and Song dynasties. When combined with our statistical analysis of the water chestnut beetle’s divergence time (113,900–126,500 years ago), we speculate that water chestnuts have existed in China for a much longer period of time than Europe. Our studies do not exclude Europe as an origin for water chestnut, because we have not systematically studied the demographic history of *T*. *natans* on a global scale. However, based on our work with the beetle and archaeological findings, we believe that the lower, central region of the Yangtze River basin is one of origin centers for *T*. *natans*. We show a proposed expansion route for the water chestnut beetle (*G*. *birmanica*) in Fig 9; furthermore, we hypothesize that the beetle likely followed the expansion and distribution of *T*. *natans* in the Late Pleistocene. Our study provides insight into the origin of water chestnut by examining population genetics of its pest, water chestnut beetle. Our results suggest that the lower, central region of the Yangtze River basin is one of the potential origin centers for the beetle, and we speculate that this dispersal was concordant with the host plant. Most importantly, we provide a plant-insect co-evolutionary system example on revealing the origin and dispersal history of a pest insect and its host.

## Supporting Information

S1 Table Sampling information. (DOC)

S2 Table Genetic diversity indices for each population. (DOC)

S3 Table Estimates of F_ST_ and gene flow (Nm) for pairs of each population based on *COI* gene. (DOC)

S4 Table Estimates of F_ST_ and gene flow (Nm) for pairs of each population based on *COII* gene. (DOC)

S5 Table Estimates of F_ST_ and gene flow (Nm) for pairs of each population based on *Cytb* gene. (DOC)
