# Supplementary material for: Genetic Structure of Water Chestnut Beetle: Providing Evidence for Origin of Water Chestnut
Source: PLoS One. 2016 Jul 26;11(7):e0159557. doi: 10.1371/journal.pone.0159557 (PMC4961436; doi:10.1371/journal.pone.0159557)
Supplement: S1 Table — (DOC) [file pone.0159557.s001.doc]

S1 Table. Sampling information.

| Region | Number | Geographical locations | Code | latitude (°N) | longitude (°E) | Number of samples | | | |
| --- | --- | --- | --- | --- | --- | --- | --- | --- | --- |
|  |  |  |  |  |  | COI | COII | Cytb | ITS2 |
| Northeastern China (NEC) | 1 | Liaoning Shenyang | SY | 41.80 | 123.43 | 4 | 6 | 5 | 5 |
|  | 2 | Liaoning Anshan | AS | 41.11 | 123.00 | 3 | 1 | 1 | 5 |
| Nouthern China (NC) | 3 | Shandong Taian | TA | 36.18 | 117.08 | 2 | 2 | 1 | 5 |
|  | 4 | Jiangsu Xuzhou | XZ | 34.27 | 117.18 | 1 | 1 | 1 | 5 |
|  | 5 | Jiangsu Huaian | HUA | 33.60 | 119.02 | 3 | 3 | 2 | 5 |
|  | 6 | Jiangsu Yancheng | YC | 33.37 | 120.14 | 2 | 1 | 1 | 5 |
|  | 7 | Jiangsu Yangzhou | YZ | 32.40 | 119.42 | 5 | 5 | 5 | 5 |
| Central China (CC) | 8 | Jiangsu Changzhou | CZ | 31.77 | 119.98 | 1 | 1 | 1 | 5 |
|  | 9 | Jiangsu Wuxi | WX | 31.56 | 120.30 | 1 | 1 | 1 | 0 |
|  | 10 | Jiangsu Suzhou | SZ | 31.31 | 120.61 | 2 | 2 | 1 | 5 |
|  | 11 | Shanghai | SH | 31.24 | 121.48 | 2 | 2 | 1 | 5 |
|  | 12 | Zhejiang Jiaxing | JX | 30.77 | 120.75 | 2 | 1 | 1 | 5 |
|  | 13 | Zhejiang Yiwu | YW | 29.30 | 120.06 | 2 | 2 | 1 | 5 |
|  | 14 | Anhui Fuyang | FY | 32.89 | 115.81 | 2 | 1 | 1 | 5 |
|  | 15 | Anhui Wuhu | WH | 31.36 | 118.38 | 2 | 2 | 1 | 5 |
|  | 16 | Hubei Xiaogan | XG | 30.92 | 113.93 | 2 | 3 | 1 | 5 |
|  | 17 | Hunan Yiyang | YY | 28.58 | 112.36 | 2 | 2 | 1 | 5 |
|  | 18 | Jiangxi Xinyu | XY | 27.82 | 114.94 | 2 | 1 | 1 | 5 |
| Southern China (SC) | 19 | Guangdong Guangzhou | GZ | 23.11 | 113.30 | 6 | 6 | 5 | 5 |
| Southweastern China (SWC) | 20 | Yunnan Baoshan | BS | 25.11 | 99.17 | 6 | 6 | 5 | 5 |
