# Supplementary material for: Genetic Structure of Water Chestnut Beetle: Providing Evidence for Origin of Water Chestnut
Source: PLoS One. 2016 Jul 26;11(7):e0159557. doi: 10.1371/journal.pone.0159557 (PMC4961436; doi:10.1371/journal.pone.0159557)
Supplement: S2 Table — (DOC) [file pone.0159557.s002.doc]

S2 Table. Genetic diversity indices for each population.

| Gene | Population | n | H | π | K |
| --- | --- | --- | --- | --- | --- |
| COI | SY | 1 | 0.00000 | 0.00000 | 0.00000 |
|  | AS | 3 | 0.00000 | 0.00000 | 0.00000 |
|  | TA | 2 | 1.00000 | 0.00478 | 4.00000 |
|  | XZ | 1 | 0.00000 | 0.00000 | 0.00000 |
|  | HUA | 2 | 0.66667 | 0.00239 | 2.00000 |
|  | YC | 2 | 1.00000 | 0.00358 | 3.00000 |
|  | YZ | 5 | 1.00000 | 0.00406 | 3.40000 |
|  | CZ | 1 | 0.00000 | 0.00000 | 0.00000 |
|  | WX | 1 | 0.00000 | 0.00000 | 0.00000 |
|  | SZ | 2 | 1.00000 | 0.00717 | 6.00000 |
|  | SH | 2 | 1.00000 | 0.00239 | 2.00000 |
|  | JX | 2 | 1.00000 | 0.00119 | 1.00000 |
|  | YW | 1 | 0.00000 | 0.00000 | 0.00000 |
|  | FY | 2 | 1.00000 | 0.00597 | 5.00000 |
|  | WH | 2 | 1.00000 | 0.00717 | 6.00000 |
|  | XG | 2 | 1.00000 | 0.00478 | 4.00000 |
|  | YY | 2 | 1.00000 | 0.00358 | 3.00000 |
|  | XY | 2 | 1.00000 | 0.00239 | 2.00000 |
|  | GZ | 3 | 0.73333 | 0.00279 | 2.33333 |
|  | BS | 4 | 0.86667 | 0.00175 | 1.46667 |
| COII | SY | 2 | 0.33333 | 0.00049 | 0.33333 |
|  | AS | 1 | 0.00000 | 0.00000 | 0.00000 |
|  | TA | 2 | 1.00000 | 0.00294 | 2.00000 |
|  | XZ | 1 | 0.00000 | 0.00000 | 0.00000 |
|  | HUA | 3 | 1.00000 | 0.00294 | 2.00000 |
|  | YC | 1 | 0.00000 | 0.00000 | 0.00000 |
|  | YZ | 2 | 0.40000 | 0.00059 | 0.40000 |
|  | CZ | 1 | 0.00000 | 0.00000 | 0.00000 |
|  | WX | 1 | 0.00000 | 0.00000 | 0.00000 |
|  | SZ | 2 | 1.00000 | 0.00294 | 2.00000 |
|  | SH | 1 | 0.00000 | 0.00000 | 0.00000 |
|  | JX | 1 | 0.00000 | 0.00000 | 0.00000 |
|  | YW | 2 | 1.00000 | 0.00734 | 5.00000 |
|  | FY | 1 | 0.00000 | 0.00000 | 0.00000 |
|  | WH | 2 | 1.00000 | 0.00734 | 5.00000 |
|  | XG | 3 | 1.00000 | 0.00685 | 4.66670 |
|  | YY | 2 | 1.00000 | 0.00441 | 3.00000 |
|  | XY | 1 | 0.00000 | 0.00000 | 0.00000 |
|  | GZ | 4 | 0.86667 | 0.00303 | 2.06667 |
|  | BS | 3 | 0.73333 | 0.00137 | 0.93333 |
| Cytb | SY | 2 | 0.40000 | 0.00087 | 0.40000 |
|  | AS | 1 | 0.00000 | 0.00000 | 0.00000 |
|  | TA | 1 | 0.00000 | 0.00000 | 0.00000 |
|  | XZ | 1 | 0.00000 | 0.00000 | 0.00000 |
|  | HUA | 2 | 1.00000 | 0.00433 | 2.00000 |
|  | YC | 1 | 0.00000 | 0.00000 | 0.00000 |
|  | YZ | 3 | 0.70000 | 0.00260 | 1.20000 |
|  | CZ | 1 | 0.00000 | 0.00000 | 0.00000 |
|  | WX | 1 | 0.00000 | 0.00000 | 0.00000 |
|  | SZ | 1 | 0.00000 | 0.00000 | 0.00000 |
|  | SH | 1 | 0.00000 | 0.00000 | 0.00000 |
|  | JX | 1 | 0.00000 | 0.00000 | 0.00000 |
|  | YW | 1 | 0.00000 | 0.00000 | 0.00000 |
|  | FY | 1 | 0.00000 | 0.00000 | 0.00000 |
|  | WH | 1 | 0.00000 | 0.00000 | 0.00000 |
|  | XG | 1 | 0.00000 | 0.00000 | 0.00000 |
|  | YY | 1 | 0.00000 | 0.00000 | 0.00000 |
|  | XY | 1 | 0.00000 | 0.00000 | 0.00000 |
|  | GZ | 3 | 0.70000 | 0.00866 | 4.00000 |
|  | BS | 2 | 0.40000 | 0.00087 | 0.40000 |
| ITS2 | SY | 4 | 0.90000 |  | 2.60000 |
|  | AS | 3 | 0.70000 |  | 2.40000 |
|  | TA | 2 | 0.40000 |  | 0.40000 |
|  | XZ | 2 | 0.40000 |  | 0.40000 |
|  | HUA | 3 | 0.70000 |  | 2.60000 |
|  | YC | 2 | 0.40000 |  | 0.40000 |
|  | YZ | 3 | 0.70000 |  | 1.60000 |
|  | CZ | 2 | 0.60000 |  | 1.20000 |
|  | SZ | 3 | 0.80000 |  | 1.40000 |
|  | SH | 3 | 0.80000 |  | 1.40000 |
|  | JX | 4 | 0.90000 |  | 1.20000 |
|  | YW | 4 | 0.90000 |  | 3.80000 |
|  | FY | 3 | 0.70000 |  | 1.20000 |
|  | WH | 4 | 0.90000 |  | 1.20000 |
|  | XG | 2 | 0.40000 |  | 0.80000 |
|  | YY | 4 | 0.90000 |  | 1.20000 |
|  | XY | 4 | 0.90000 |  | 3.00000 |
|  | GZ | 3 | 0.70000 |  | 4.20000 |
|  | BS | 2 | 0.40000 |  | 0.40000 |

H: Haplotype diversity; π: nucleotide diversity; K: average number of nucleotide differences.
