# Supplementary material for: Genetic Structure of Water Chestnut Beetle: Providing Evidence for Origin of Water Chestnut
Source: PLoS One. 2016 Jul 26;11(7):e0159557. doi: 10.1371/journal.pone.0159557 (PMC4961436; doi:10.1371/journal.pone.0159557)
Supplement: S4 Table — (DOC) [file pone.0159557.s004.doc]

S4 Table. Estimates of F_ST_ and gene flow (N_m_) for pairs of each population based on COII gene.


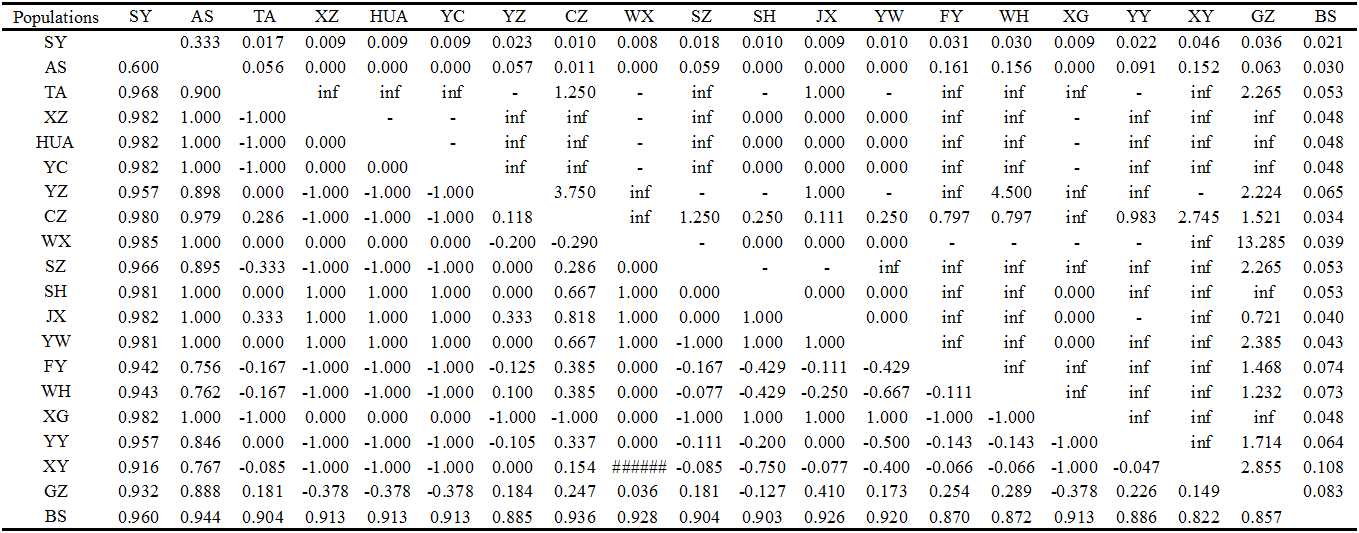


The data above the diagonal are N_m_; the data below the diagonal are F_ST_.
